# Trends in Maternal Death Post-*Dobbs v Jackson Women’s Health*

**DOI:** 10.1001/jamanetworkopen.2024.30035

**Published:** 2024-08-27

**Authors:** Amanda Jean Stevenson, Leslie Root

**Affiliations:** 1 University of Colorado Population Center, University of Colorado Boulder; 2 Institute of Behavioral Science, University of Colorado Boulder; 3 Department of Sociology, University of Colorado Boulder

## Abstract

This cross-sectional study examines monthly maternal deaths after the Dobbs v Jackson Women’s Health decision.

## Introduction

After the *Dobbs v Jackson Women’s Health* decision struck down *Roe v Wade* in June 2022, half of US states began enforcing new restrictions on abortion, and 14 banned abortion completely or with rare exceptions. Scholars hypothesize that abortion bans increase maternal mortality,^1,2^ but supporters of abortion bans claim that maternal death has declined since *Dobbs v Jackson Women’s Health*,^3^ pointing to precipitous declines in a key national maternal death statistic after the ruling.^4^

The National Center for Health Statistics (NCHS) publishes annual national maternal mortality rates and reflects the flow of mortality data by also publishing monthly 12-month ending provisional maternal mortality counts more frequently. Each month’s 12-month count is the sum of maternal deaths in the 12-month period ending that month, combining final and provisional data as necessary. For example, the June 2021 count includes all maternal deaths July 1, 2020, to June 30, 2021. These monthly sums dropped precipitously after *Dobbs v Jackson Women’s Health.* We use monthly maternal deaths and monthly COVID-19 deaths to examine the observed decline in the 12-month ending counts.

## Methods

This cross-sectional study was determined not to involve human participants by the University of Colorado institutional review board and followed the Strengthening the Reporting of Observational Studies in Epidemiology (STROBE) reporting guideline. For the months when data are available, we reported monthly counts of maternal deaths from final (2018 to 2021) and provisional (2022 to 2023) mortality surveillance^5^ and provisional counts of deaths involving COVID-19 among women of reproductive age (15 to 44 years), January 2020 to September 2023,^6^ both from US Centers for Disease Control and Prevention (CDC). We decomposed monthly change in the 12-month sums of maternal deaths. Change in the sum depends on deaths in the current month and deaths 12 months ago, each of which contributes change equal to the difference between the deaths in that month and the average monthly deaths in the prior 12-month ending sum (eAppendix in Supplement 1). The data were observational. Analyses took place in April 2024 using Excel version 16.83 (Microsoft).

## Results

**Figure 1.**
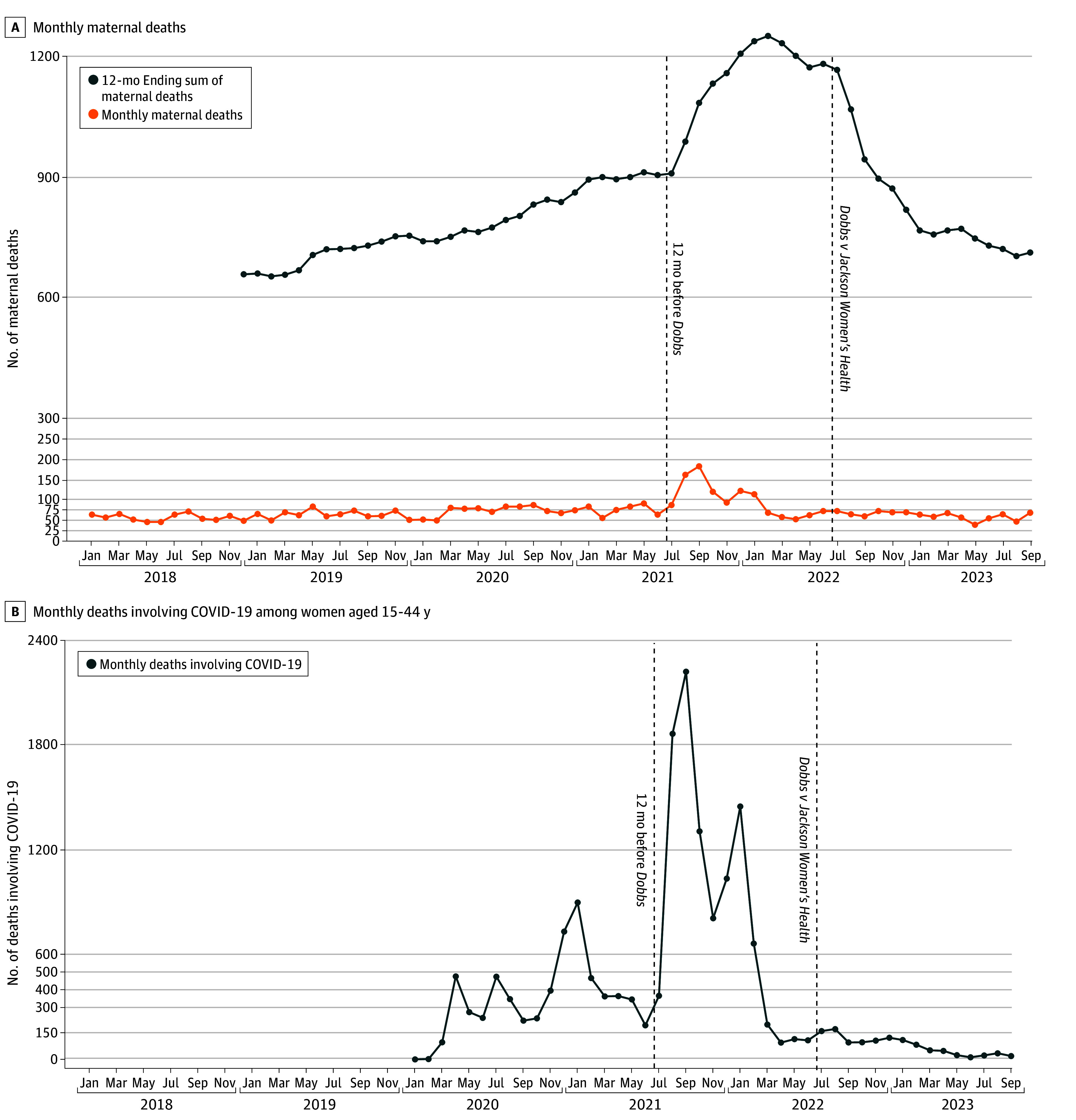
Monthly 12-Month Ending Provisional Counts of Maternal Deaths, Monthly Maternal Deaths, and Monthly Deaths Involving COVID-19 Among Women Aged 15 to 44 Years Panels A and B have differing y-axes. Vertical lines indicate *Dobbs v Jackson Women’s Health* decision and 12 months before. Differences in periods displayed reflect underlying data availability.

**Figure 2.**
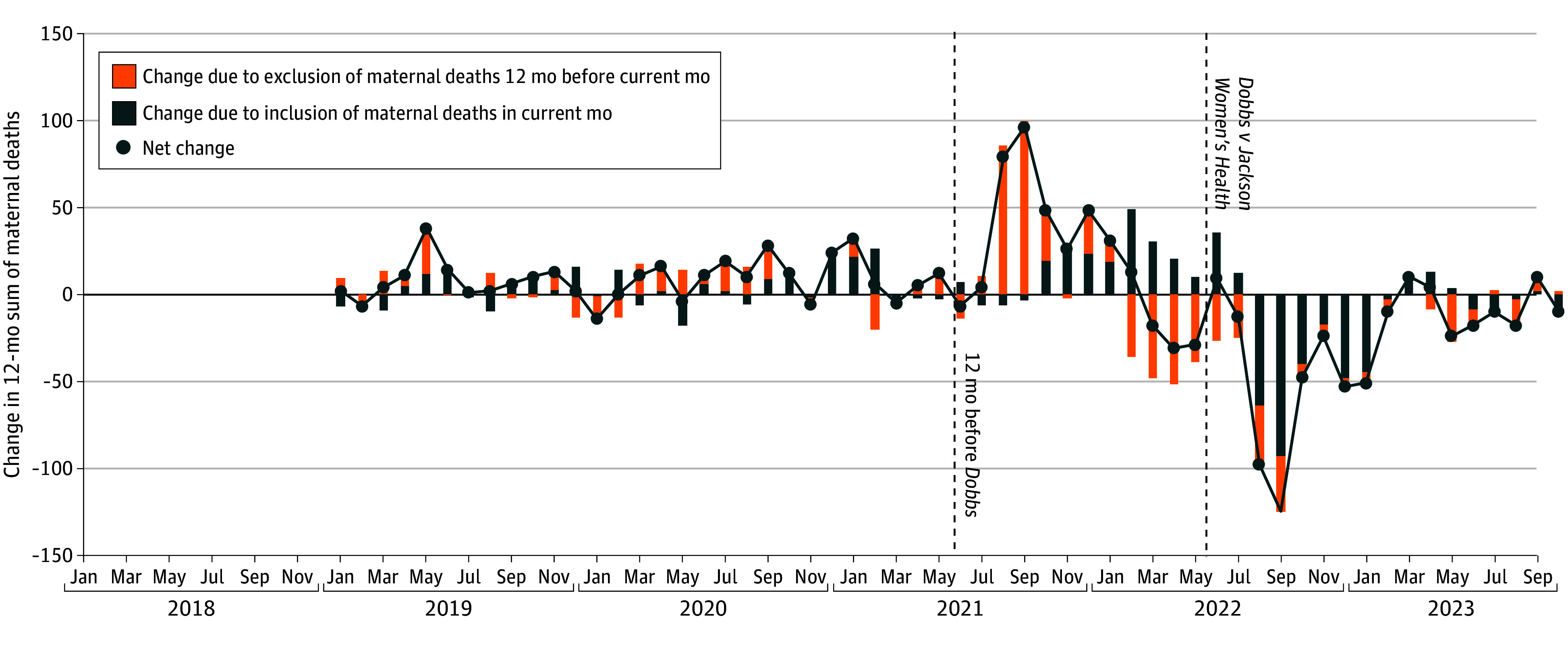
Components of Month-Over-Month Change in 12-Month Ending Sum of Maternal Deaths

We observed 4802 maternal deaths January 2018 to September 2023 (mean [SD] monthly deaths, 69.6 [24.2] monthly deaths). Monthly deaths range from 37 deaths in May 2023 to 182 deaths in September 2021. From August 2022 to January 2023, 12-month ending sums of maternal deaths declined 28.2% from 1069 to 768 deaths. Meanwhile, monthly maternal deaths were stable, beginning at 63 deaths and ending at 62 deaths (mean [SD] monthly deaths, 65.2 [5.1] monthly deaths). Corresponding with a spike in COVID-19 deaths, monthly maternal deaths had been elevated during the corresponding period 1 year earlier from August 2021 to January 2022 (mean [SD] monthly deaths, 115.7 [33.4] monthly deaths) (Figure 1). These elevated death counts moved through the 12-month ending sum as a large positive shock, which drove the sum up as they entered from 908 in July 2021 (the month before deaths rose abruptly in August 2021) to 1236 in January 2022 and drove the sum down as they exited from 1167 in July 2022 to 768 in January 2023 (Figure 2). For example, of the 125-death decline in the 12-month sum between August and September 2022, a 92.9-death decline (74%) was due to excluding August deaths from the sum.

## Discussion

In this study, decline in the NCHS 12-month ending maternal death sums after *Dobbs v Jackson Women’s Health* appeared to be driven by elevated levels of maternal death during the 2021 spike in COVID-19–related deaths rather than changes after *Dobbs v Jackson Women’s Health*. For example, 74% of the 125-death decline in the 12-month sum between August and September 2022 was caused by excluding August 2021 deaths from the September 12-month sum. Similarly high proportions of declines September 2022 to February 2023 are explained by deaths 12 months before. Declines in the 12-month sums do not provide evidence that maternal deaths decreased after *Dobbs v Jackson Women’s Health.* Our study is limited by its descriptive design and reliance on maternal death enumeration via death certificates.

This spike in maternal deaths in the second half of 2021 affects the 12-month sum of maternal deaths in 2022. In general, trends in moving summaries like these reflect changes on both ends of their periods; circumspection is required when they are used for policy evaluation. Given the heterogeneous timing and scope of abortion bans and the many processes by which they may influence maternal health, we must clearly articulate the limitations of available measures and specify when and how impacts may be observed.

## Notes

Supplement 1. **eAppendix. **Technical Appendix

Supplement 2.
Data Sharing Statement
