# Supplementary material for: Trends in Maternal Death Post-Dobbs v Jackson Women’s Health
Source: JAMA Netw Open. 2024 Aug 27;7(8):e2430035. doi: 10.1001/jamanetworkopen.2024.30035 (PMC11350468; doi:10.1001/jamanetworkopen.2024.30035)
Supplement: Supplement 1. — eAppendix. Technical Appendix [file jamanetwopen-e2430035-s001.pdf]

Supplemental Online Content

Stevenson AJ, Root L. Trends in maternal death post-*Dobbs*. *JAMA Netw Open*. 2024;7(8):e2430035. doi:10.1001/jamanetworkopen.2024.30035

eAppendix. Technical Appendix

This supplemental material has been provided by the authors to give readers additional information about their work.

© 2024 Stevenson AJ et al. *JAMA Network Open*.

eAppendix. Technical Appendix

This appendix provides more details about 12-month ending sums of maternal deaths and how we describe the sources of changes in the sums in Figure 2.

The National Center for Health Statistics calculates the 12-month ending sum for each month by adding all maternal deaths in the 12-month period ending in the month for which the sum is calculated. In other words, in month m the 12-month ending sum (S_m) is the sum of deaths in month m and the prior 11 months:

(1) $$S_m = \sum_{i=m-11}^{m} deaths_i$$

Figure 2 displays the month-over-month absolute change in 12-month ending sums of maternal deaths and the contributions to the absolute change due to the month leaving the sum and the month entering the sum.

And the absolute change in the sum from S_{m-1} to S_m may be expressed as the difference between deaths in the month entering the sum ($deaths_m$) and deaths in the month leaving the sum ($deaths_{m-12}$):

(2) $$S_m - S_{m-1} = \sum_{i=m-11}^{m} deaths_i - \sum_{i=m-12}^{m-1} deaths_i = deaths_m - deaths_{m-12}$$

To describe the relative contributions of the entrance of $deaths_m$ and the exit of $deaths_{m-12}$ to the difference between S_m and S_{m-1}, we subtract the average monthly deaths in months $m-11$ through $m-1$, $\frac{S_{m-1}}{12}$ for each term in the right hand side of Equation 2. In this way, each term of the difference becomes an expression of how

© 2024 Stevenson AJ et al. *JAMA Network Open*.

much the sum would change if the other element was equal to the average monthly deaths in S_{m-1}.

(3) $$S_m - S_{m-1} = \left[deaths_m - \frac{S_{m-1}}{12}\right] - \left[deaths_{m-12} - \frac{S_{m-1}}{12}\right]$$

Figure 2 plots the net month-over-month change in the 12-month ending count as the green connected line and the contributions of change from the two changes in the months included in the sum as stacked bars. The change due to the month gained (the first term on the right hand side of Equation 3, $\left[deaths_m - \frac{S_{m-1}}{12}\right]$) is displayed as orange bars, and the change due to the month lost (the second term on the right had side of Equation 3, $-\left[deaths_{m-12} - \frac{S_{m-1}}{12}\right]$) is displayed as blue bars.

The change in the sum from month $m-1$ to month m is the difference between the sum in month $m-1$ (S_{m-1}) and the sum in month m (S_m). Both S_{m-1} and S_m include deaths in months $m-11$ through $m-1$. So the difference $S_{m-1} - S_m$ is generated jointly by (1) the fact that deaths in month $m-12$ are included in S_{m-1} and not in S_m and (2) that deaths in month m are included in S_m but not in S_{m-1}.

© 2024 Stevenson AJ et al. *JAMA Network Open*.
