# Supplementary material for: Trends in Maternal Death Post-Dobbs v Jackson Women’s Health
Source: JAMA Netw Open. 2024 Aug 27;7(8):e2430035. doi: 10.1001/jamanetworkopen.2024.30035 (PMC11350468; doi:10.1001/jamanetworkopen.2024.30035)
Supplement: Supplement 2. — Data Sharing Statement [file jamanetwopen-e2430035-s002.pdf]

Data Sharing Statement

Data
Data available: Yes
Data types: Data (not involving human participants)
How to access data: Underlying cause of death ICD 10 codes A34, O00–O95, and O98–O99, 2018-2021: https://wonder.cdc.gov/controller/saved/D158/D383F806 Same codes for pregnancy as underlying cause in provisional data: https://wonder.cdc.gov/controller/saved/D176/D383F816 CDC Provisional COVID-19 Deaths by Sex and Age: https://data.cdc.gov/d/9bhg-hcku
When available: With publication

Supporting Documents
Document types: None

Additional Information
Who can access the data: All data are publicly available on the web.
Types of analyses: The data are freely available to all who consent to data use restrictions.
Mechanisms of data availability: Investigators have no control over who may access the data as they are controlled by Federal agencies and made available by those agencies via listed websites.
